# Atmospheric corrosion of metals in industrial city environment

**DOI:** 10.1016/j.dib.2015.02.017

**Published:** 2015-03-04

**Authors:** Elzbieta Kusmierek, Ewa Chrzescijanska

**Affiliations:** Lodz University of Technology, Department of Chemistry, Institute of General and Ecological Chemistry, ul. Zeromskiego 116, 90-924 Lodz, Poland

**Keywords:** Atmospheric corrosion, Industrial city environment, Metals, Corrosion potential, Surface morphology

## Abstract

Atmospheric corrosion is a significant problem given destruction of various materials, especially metals. The corrosion investigation in the industrial city environment was carried out during one year exposure. Corrosion potential was determined using the potentiometric method. The highest effect of corrosion processes was observed during the winter season due to increased air pollution. Corrosion of samples pre-treated in tannic acid before the exposure was more difficult compared with the samples without pretreatment. The corrosion products determined with the SEM/EDS method prove that the most corrosive pollutants present in the industrial city air are SO_2_, CO_2_, chlorides and dust.

**Specifications table**

**Table:** 

Subject area	*Chemistry*
More specific subject area	*Corrosion of metals*
Type of data	*Tables*, *figures*
How data was acquired	*Potentiometric measurements were performed using μAutolab potentiostat*/*galvanostat*, *SEM*/*EDS analysis*
Data format	*Analysed*
Experimental factors	*Metal samples were mechanically grounded with abrasive paper*, *rinsed with distilled water*, *degreased with aceton and dried in air*
Experimental features	*Potentiometric curves were recorded in a three-electrode cell, morphology of sample surface was investigated by the SEM*/*EDS method*
Data source location	*Lodz*, *Poland*
Data accessibility	*The data is with this article*

## Value of the data

• Changes in corrosion potential were related to atmospheric conditions.
• Surface of metal samples was characterised after the exposition to industrial city environment by SEM/EDS analysis.
• Tannic acid was applied as corrosion inhibitor before exposition to the environment.

## 1 Data, experimental design, materials and methods

### 1.1 Sample preparation

Corrosion investigations were carried out with the application of metal samples: iron, industrial copper and zinc samples. Surface area of metal samples exposed to performance of atmospheric corrosion factors was 2 cm^2^. Prior to the measurements, each sample was mechanically grounded with 600 grade of abrasive paper, rinsed with distilled water, degreased in acetone (CH_3_COCH_3_) of analytical grade and dried in air.

### 1.2 Electrochemical studies and surface analysis

Electrochemical measurements were carried out using a three-electrode cell with Pt counter electrode and saturated calomel electrode (SCE) as a reference electrode. Surface area of metal samples exposed to atmospheric corrosion was 2 cm^2^. An electrical contact to the metal sample was made of the same metal as a sample and was inserted in a Teflon tube in order to insulate it from the solution. Potentiometric curves were recorded in μAutolab electroanalytical instrument (Metrohm-EcoChemie, The Netherlands). In order to estimate a change in the susceptibility of tested metals and alloys to atmospheric corrosion, their corrosion potential (*E*_corr_) was measured in specially prepared solution in open circuit for 30 min. The solution was prepared using distilled water with additions of the following chemicals: NaCl (30 mg dm^−3^), Na_2_SO_4_ (30 mg dm^−3^) and NaHCO_3_ (30 mg dm^−3^) and was naturally aerated in order to simulate the industrial city environment. Changes in the susceptibility to corrosion can be caused not only by varying meteorological conditions and pollution of air but also by coverage of tested metal and alloys with various corrosion products.

Surface of metal samples exposed to the industrial city environment was characterised morphologically using scanning electron microscopy SEM (S-4700 Hitachi) coupled with energy dispersive X-ray analysis EDS (Thermo Noran).

### 1.3 Field exposure

Tests were carried out at one site located in the centre of the city (Fig. S1) near power and heating plant. The test site was characterised by compact settlement which limited traffic due to narrow streets and also significantly limited quick ventilation of the area. The main pollutants in Lodz air included SO_2_, NO_2_, CO, PM_10_ and PM_2.5_ [1]. Total emission of these pollutants in Lodz province and Lodz agglomeration is presented in Table 1. Lodz agglomeration has surface area of 1836.2 km^2^ which is almost an order of magnitude lower than the surface area of Lodz province. Lodz agglomeration includes Lodz city and small adjacent towns. PM_10_ and PM_2.5_ refers to particles with diameters of 10 and 2.5 nm or less, respectively. These particles deposit at metal surfaces and absorb moisture. The moisture layer on the metal surfaces contains dissolved NO_2_, SO_2_ and CO_2_, and forms the electrolyte film in which corrosion processes proceed with electrochemical mechanism.

Metal samples were exposed to the industrial city environment for one year starting in September 2011. Prior to the exposure, one set of metal samples exposed to the environment in the city centre was immersed in tannic acid (ACS reagent, Sigma-Aldrich, 100 mg dm^−3^) for 5 min. Next the samples were dried in air under room temperature. Measurements of *E*_corr_ by the potentiometric method, were carried out at least once every two weeks. Results of the measurements were related with changes in the atmospheric conditions in Lodz agglomeration over the period of the studies.

### 1.4 Atmospheric corrosion vs. atmospheric pollution

Atmospheric corrosion of metals and their alloys is very common in the industrial city environment due to the high concentration of corrosive pollutants in air. It results in destruction of various materials, especially metals and their alloys. The contribution of the atmospheric corrosion in the overall corrosion costs is very high [2,3]. In 2010, annual losses caused by the corrosion in Poland were estimated as 8% of GDP (gross domestic product) [4]. However, in Western European countries, this indicator is at the level of 3–5% of GDP [5]. Atmospheric corrosion in Lodz agglomeration is caused by point, linear and area sources of air pollution [1,6,7]. Thus, protection against corrosion is very important. One of protection methods includes application of corrosion inhibitors, especially green inhibitors [8]. Tannic acid can be applied as a corrosion inhibitor. Its inhibitive action towards corrosion of the tested metals was proved in our previous paper [9].

Two sets of metal samples were exposed to the environment in one site located in the centre of Lodz agglomeration. This location is specific due to increasing pollutants emissions in winter. Unfavourable meteorological conditions and air pollution are the reason why corrosive power of the atmosphere in the centre of Lodz increases in winter and sometimes acceptable values for gaseous pollutants concentrations in air are exceeded. Changes in the atmospheric conditions as well as in concentration of main pollutants in air within a period of the exposure are presented in Figs. S2 and S3.

**Fig. 1:**
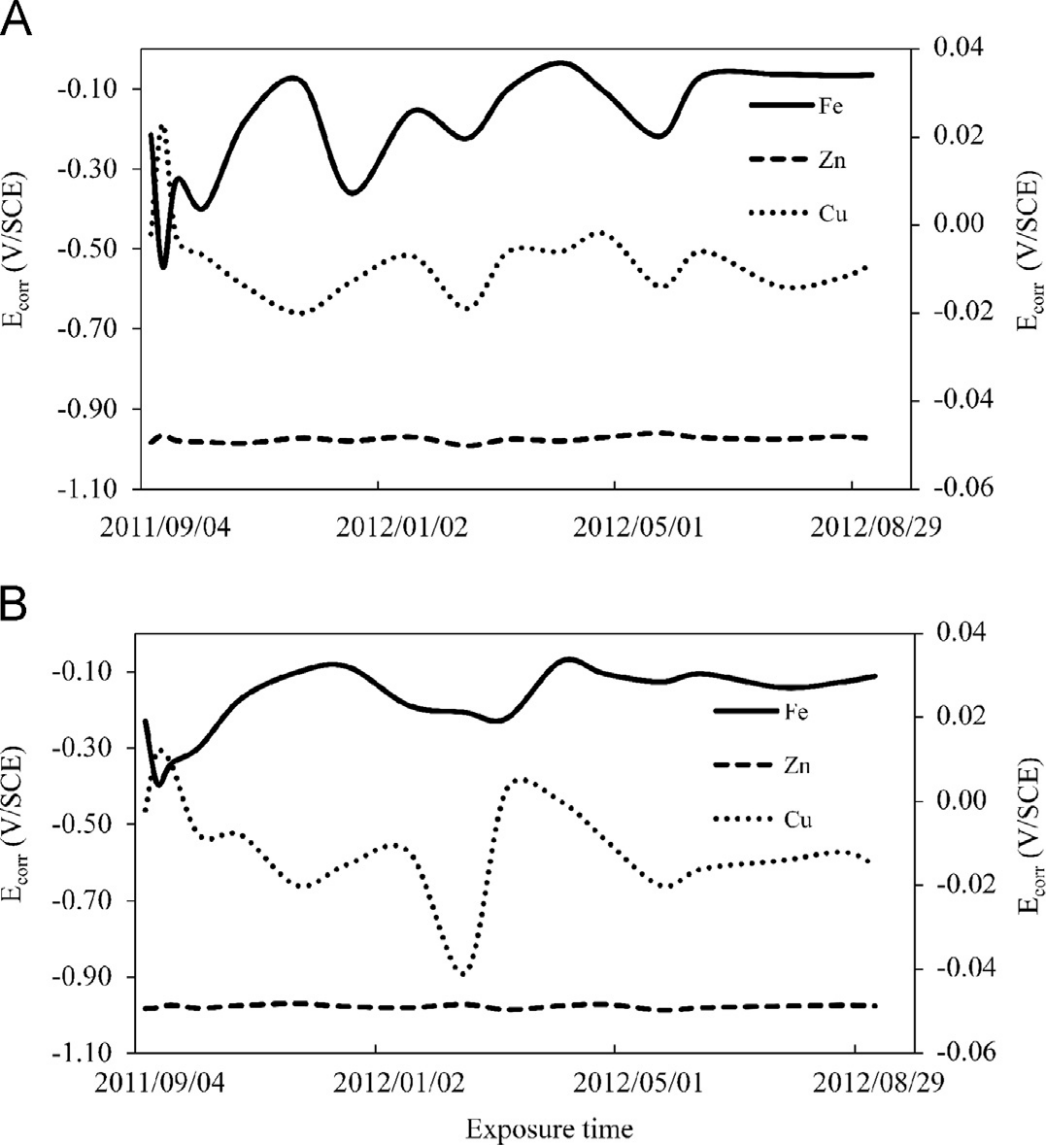
Changes in *E*_corr_ of metal samples exposed to the environment in the centre of Lodz agglomeration without (A) and with immersion in tannic acid solution before exposure (B), Fe and Zn – left axis, Cu – right axis.

In the specified periods, *E*_corr_ was measured for all exposed samples using potentiometric method. Changes in *E*_corr_ values were related to the atmospheric conditions (temperature and humidity) and air pollution within the period of the investigation. Results of *E*_corr_ determination are presented in Fig. 1A. Value of *E*_corr_ determined for Fe increased from −0.215 to −0.065 V during the exposure time but were almost stable towards the end. The value of *E*_corr_ did not increase any further because coverage of the Fe sample with the corrosion products partially prevented further corrosion processes. However, during the first week of the exposure a significant decrease in *E*_corr_ to −0.546 V was observed. During that time, Fe sample intensively corroded because its surface carefully cleaned prior to the exposure, was quickly covered with dust and moisture layer. Later an increase in *E*_corr_ to −0.08 V was observed due to the partial coverage of Fe surface with the corrosion products. At the beginning of January, there was subsequent decrease in *E*_corr_ to −0.36 V resulting from an increase in humidity and concentration of SO_2_, NO_2_ and dust in air (Figs. S2 and S3). During the winter season, Fe samples corroded easier than in the spring–summer season.

In the case of Zn samples, the effect of the industrial city environment on their corrosion was very slight. The values of *E*_corr_ for this metal were the lowest in comparison with other samples and this means that Zn corroded the easiest taking into consideration thermodynamic aspect. *E*_corr_ changed within the range of −0.985 to −0.965 V but without any clear increases or decreases.

*E*_corr_ values determined for Cu samples fluctuated during the exposure period. At the end of the exposure period, *E*_corr_ was ca. 6 mV lower than at the beginning. However, during this time changes in *E*_corr_ were clear. During the first week, *E*_corr_ first increased and then decreased. Surface of Cu samples was quickly covered with the corrosion products which partially prevented further corrosion. Next, *E*_corr_ decreased due to unfavourable atmospheric conditions and air pollution. Starting from the end of November, *E*_corr_ increased, even though the atmospheric conditions were not more favourable and air pollution did not decrease. Probably, Cu samples surface was covered with oxides during the previous period and these oxides partially prevented further corrosion, although they did not stop it. Starting from the half of February, a slight decrease in *E*_corr_ was observed due to an increase in air pollution, especially in SO_2_ concentration in air (Figs. S3 and 1A).

Metal samples, immersed in tannic acid solution before the exposure showed a little bit different changes in *E*_corr_. In the case of Fe, *E*_corr_ decreased by ca. 0.17 V during the first week. Afterwards, *E*_corr_ increased but did not undergo so significant changes as in the case of Fe samples which did not undergo pre-treatment in the tannic acid. However, at the end of the exposure period, *E*_corr_ was about 60 mV lower than in the case of Fe samples not pretreated. This means that Fe samples were partially protected against corrosion till the beginning of April. In the subsequent period, a protective film which formed in the reaction of the corrosion products with the tannic acid, was probably rinsed out with rain or snow. Thus, Fe samples should have been immersed in the tannic acid solution at least several times during the exposure period in order to efficiently prevent corrosion processes. In the case of Zn samples, no significant changes in *E*_corr_ were observed. Their immersion in the tannic acid did not give any positive results. Cu samples showed an increase in *E*_corr_ during the first exposure week followed by significant fluctuations of its value. In the case of Cu, *E*_corr_ decreased to −0.015 V in the winter season (Fig. 1B). This fact can be explained by an increase in humidity and concentration of air pollutants at this time (Figs. S2 and S3). At the end of the exposure period, *E*_corr_ value for Cu samples was about 13 mV lower than before the exposure.

However, one immersion of all metal samples in the tannic acid prior to the exposure was certainly not enough for efficient prevention of the corrosion processes. The immersions should have been repeated periodically several times during the exposure period.

### 1.5 Morphological studies of metal samples surfaces

The surface of metal samples was examined before and after exposure to the industrial city environment using SEM electron scanning microscopy coupled with the energy dispersive X-ray analysis EDS. Appearance of the metal samples after the exposure proved their coverage with corrosion products which resulted in the change in colour and structure of the surface.

Fe samples showed clear change in colour of the surface due to coverage with rust. Scanning electron micrographs recorded after the exposure (Fig. S4) demonstrate the presence of various corrosion products. There are round shaped agglomerates with a diameter of about 25 μm visible at the surface. They can correspond to one of the corrosion products, e.g., γ-FeOOH (lepidocrocite). EDS analysis results showed the presence of O and S at the Fe sample surface after the exposure. This indicates that the surface was covered with different oxides such as: FeOOH, Fe_2_O_3_, Fe_3_O_4_ as well as with corrosion products containing sulphur like FeSO_4_. The presence of particulate matter (Si and Al) proves the coverage of the surface with dust which absorbs moisture and accelerates corrosion processes.

Appearance of Cu sample after one year exposure demonstrates significant change in colour of its surface and significant corrosion. Scanning electron micrographs show that the surface of Cu sample is covered with successive layers of corrosion products coating particulate matters (Fig. S5). Results of EDS analysis prove the presence of O, C, S, Al, Si and Cl. Atmospheric corrosion of Cu samples can result in its patination due to covering with basic carbonate and hydrocarbonate of copper. Moreover, such corrosion products as CuO, Cu_2_O and Cu(OH)_2_ can be formed. If air contains SO_2_, then the formation of CuSO_4_ and Cu(OH)_2_ mixture is possible. During the winter season, chlorides can be present in air due to application of KCl or NaCl in order to prevent formation of ice on sidewalks and roads in the city. Thus, formation of Cu_2_(OH)_3_Cl is possible in the presence of Cl^−^. The formation of above mentioned corrosion products is confirmed by EDS analysis.

Zn samples exposed to the industrial city environment were covered with steel-grey layer of corrosion products. In the first step of corrosion processes, Zn(OH)_2_ is formed and is converted to CaCO_3_·3Zn(OH)_2_ as a result of CO_2_ presence in air. If SO_2_ is present in air then corrosion processes of Zn are accelerated and formation of different corrosion products like ZnSO_3_, ZnSO_4_ and ZnSO_4_·*n*H_2_O is possible. After one year exposure of Zn samples, SEM images (Fig. S6) demonstrate the layer of corrosion products consisting of small round shaped agglomerates with a diameter of 10 μm or less. During atmospheric corrosion, Zn often undergoes pitting, especially if chlorides are present in air. EDS analysis confirmed the presence of C, S, O, Al, Ca, Si, Fe and Cl on Zn sample surface. Such combination of elements is characteristic for industrial areas. In the case of Zn, the most aggressive component of air is CO_2_ and SO_2_.

Exposure of metal samples to the industrial city environment for one year showed that the highest effect of corrosion processes was observed during the winter season. It resulted from higher air pollution at this time caused by the neighbouring power and heating plant, heavy automotive traffic, increasing emission of air pollutants by furnaces and other types of heating, and high-density housing limiting air flow in the area. Corrosion potential of metal samples pre-treated in the tannic acid solution before the exposure did not fluctuate so much over the exposure period as it was observed for samples not-pretreated. However, only one immersion in the tannic acid appeared to be not enough for efficient prevention of the atmospheric corrosion. It should have been repeated several times during the exposure.

Analysis of the samples׳ surface after the exposure to the industrial city environment proved their advanced corrosion except for copper. Results of metal surface morphology studies show that the highest impact on the corrosion processes was found in the case of SO_2_, dust, humidity and CO_2_. These pollutants concentrations reached the highest values during the winter season. The presence of chlorides in air during the winter season, also influenced corrosion processes. Combination of elements determined at the metal surfaces was typical for industrial areas.

## Figures and Tables

**Table 1:** Total emission of selected pollutants in Lodz region and Lodz agglomeration in 2011 [1].

Pollutant (Mg year^−1^)	Area	
	Lodz province	Lodz agglomeration
SO_2_	108 615.29	11 805.06
NO_2_	114 068.13	10 128.69
CO	112 663.38	27 105.41
PM_10_	46 373.19	6 489.05
PM_2.5_	20 296.13	2 827.16
