# Supplementary material for: Atmospheric corrosion of metals in industrial city environment
Source: Data Brief. 2015 Mar 4;3:149–54. doi: 10.1016/j.dib.2015.02.017 (PMC4510135; doi:10.1016/j.dib.2015.02.017)
Supplement: Supplementary file 1 — Supplementary Material [file mmc1.zip › Supplementary materials.docx]

**Supplementary materials**

**Fig. S1.** Map of Lodz Province, Agglomeration and City with location of test site.

**Fig. S2.** Total emission of selected pollutants in Lodz Province over the period of 2007-2011 and in Lodz agglomeration in 2011 [1].

**Fig. S3.** Changes in the atmospheric conditions and air pollution in the centre of Lodz agglomeration within the period of the exposure; (A) temperature and humidity, (B) PM_10_ dust concentration, (C) SO_2_ and NO_2_ concentration.

**Fig. S4.** SEM/EDS analysis results obtained on the surface of Fe samples after one year exposure to the industrial city environment in Lodz agglomeration.

**Fig. S5.** SEM/EDS analysis results obtained on the surface of Cu samples after one year exposure to the industrial city environment in Lodz agglomeration.

**Fig. S6.** SEM/EDS analysis results obtained on the surface of Zn samples after one year exposure to the industrial city environment in Lodz agglomeration.
